# Supplementary material for: Methodological considerations in assessment of language lateralisation with fMRI: a systematic review
Source: PeerJ. 2017 Jul 11;5:e3557. doi: 10.7717/peerj.3557 (PMC5508809; doi:10.7717/peerj.3557)
Supplement: Appendix S1 [file peerj-05-3557-s001.doc]

**List of 76 selected studies**

1. Abbott, D. F., Waites, A. B., Lillywhite, L. M., & Jackson, G. D. (2010). fMRI assessment of language lateralization: An objective approach. *Neuroimage, 50*(4), 1446-1455. doi:10.1016/j.neuroimage.2010.01.059
2. Adcock, J., Wise, R., Oxbury, J., Oxbury, S., & Matthews, P. (2003). Quantitative fMRI assessment of the differences in lateralization of language-related brain activation in patients with temporal lobe epilepsy. *Neuroimage, 18*(2), 423-438. doi:10.1016/S1053-8119(02)00013-7
3. Allendorfer, J. B., Hernando, K. A., Hossain, S., Nenert, R., Holland, S. K., & Szaflarski, J. P. (2016). Arcuate fasciculus asymmetry has a hand in language function but not handedness. *Human Brain Mapping, 37*(9), 3297-3309. doi:10.1002/hbm.23241
4. Baciu, M., Juphard, A., Cousin, E., & Le Bas, J. (2005). Evaluating fMRI methods for assessing hemispheric language dominance in healthy subjects. *European Journal of Radiology, 55*(2), 209-218. doi:10.1016/j.ejrad.2004.11.004
5. Backes, W., Deblaere, K., Vonck, K., Kessels, A., Boon, P., Hofman, P., et al. (2005). Language activation distributions revealed by fMRI in post-operative epilepsy patients: Differences between left- and right-sided resections. *Epilepsy Research, 66*(1-3), 1-12. doi:10.1016/j.eplepsyres.2005.06.007
6. Berl, M. M., Zimmaro, L. A., Khan, O. I., Dustin, I., Ritzl, E., Duke, E. S., et al. (2014). Characterization of atypical language activation patterns in focal epilepsy. *Annals of Neurology, 75*(1), 33-42. doi:10.1002/ana.24015
7. Bethmann, A., Tempelmann, C., De Bleser, R., Scheich, H., & Brechmann, A. (2007). Determining language laterality by fMRI and dichotic listening. *Brain Research, 1133*(1), 145-157. doi:10.1016/j.brainres.2006.11.057
8. Binder, J. R., Swanson, S. J., Hammeke, T. A., & Sabsevitz, D. S. (2008). A comparison of five fMRI protocols for mapping speech comprehension systems. *Epilepsia, 49*(12), 1980-1997. doi:10.1111/j.1528-1167.2008.01683.x
9. Brennan, N. M. P., Whalen, S., Branco, D. d. M., O'Shea, J. P., Norton, I. H., & Golby, A. J. (2007). Object naming is a more sensitive measure of speech localization than number counting: Converging evidence from direct cortical stimulation and fMRI. *Neuroimage, 37*, S100-S108. doi:10.1016/j.neuroimage.2007.04.052
10. Clements, A. M., Rimrodt, S. L., Abel, J. R., Blankner, J. G., Mostofsky, S. H., Pekar, J. J., et al. (2006). Sex differences in cerebral laterality of language and visuospatial processing. *Brain and Language, 98*(2), 150-158. doi:10.1016/j.bandl.2006.04.007
11. Cousin, E., Peyrin, C., Pichat, C., Lamalle, L., Le Bas, J., & Baciu, M. (2007). Functional MRI approach for assessing hemispheric predominance of regions activated by a phonological and a semantic task. *European Journal of Radiology, 63*(2), 274-285. doi:10.1016/j.ejrad.2007.01.030
12. Deblaere, K., Backes, W., Hofman, P., Vandemaele, P., Boon, P., Vonck, K., et al. (2002). Developing a comprehensive presurgical functional MRI protocol for patients with intractable temporal lobe epilepsy: A pilot study. *Neuroradiology, 44*(8), 667-673. doi:10.1007/s00234-002-0800-4
13. Dodoo-Schittko, F., Rosengarth, K., Doenitz, C., & Greenlee, M. W. (2012). Assessing language dominance with functional MRI: The role of control tasks and statistical analysis. *Neuropsychologia, 50*(11), 2684-2691. doi:10.1016/j.neuropsychologia.2012.07.032
14. Doucet, G. E., Pustina, D., Skidmore, C., Sharan, A., Sperling, M. R., & Tracy, J. I. (2015). Resting-state functional connectivity predicts the strength of hemispheric lateralization for language processing in temporal lobe epilepsy and normals. *Human Brain Mapping, 36*(1), 288-303. doi:10.1002/hbm.22628
15. Drager, B., Jansen, A., Bruchmann, S., Forster, A., Pleger, B., P'Zwitserlood, et al. (2004). How does the brain accommodate to increased task difficulty in word finding? A functional MRI study. *Neuroimage, 23*(3), 1152-1160. doi:10.1016/j.neuroimage.2004.07.005
16. Fernandez, G., de Greiff, A., von Oertzen, J., Reuber, M., Lun, S., Klaver, P., et al. (2001). Language mapping in less than 15 minutes: Real-time functional MRI during routine clinical investigation. *Neuroimage, 14*(3), 585-594. doi:10.1006/nimg.2001.0854
17. Fesl, G., Bruhns, P., Rau, S., Wiesmann, M., Ilmberger, J., Kegel, G., et al. (2010). Sensitivity and reliability of language laterality assessment with a free reversed association task-a fMRI study. *European Radiology, 20*(3), 683-695. doi:10.1007/s00330-009-1602-4
18. Gaillard, W., Balsamo, L., Xu, B., Grandin, C., Braniecki, S., Papero, P., et al. (2002). Language dominance in partial epilepsy patients identified with an fMRI reading task. *Neurology, 59*(2), 256-265.
19. Gaillard, W., Sachs, B., Whitnah, J., Ahmad, Z., Balsamo, L., Petrella, J., et al. (2003). Developmental aspects of language processing: FMRI of verbal fluency in children and adults. *Human Brain Mapping, 18*(3), 176-185. doi:10.1002/hbm.10091
20. Haeberling, I. S., Badzakova-Trajkov, G., & Corballis, M. C. (2011). Callosal tracts and patterns of hemispheric dominance: A combined fMRI and DTI study. *Neuroimage, 54*(2), 779-786. doi:10.1016/j.neuroimage.2010.09.072
21. Haeberling, I. S., Steinemann, A., & Corballis, M. C. (2016). Cerebral asymmetry for language: Comparing production with comprehension. *Neuropsychologia, 80*, 17-23. doi:10.1016/j.neuropsychologia.2015.11.002
22. Harrington, G., Buonocore, M., & Farias, S. (2006). Intrasubject reproducibility of functional MR imaging activation in language tasks. *American Journal of Neuroradiology, 27*(4), 938-944.
23. Hernandez, N., Andersson, F., Edjlali, M., Hommet, C., Cottier, J. P., Destrieux, C., et al. (2013). Cerebral functional asymmetry and phonological performance in dyslexic adults. *Psychophysiology, 50*(12), 1226-1238. doi:10.1111/psyp.12141
24. Hund-Georgiadis, M., Lex, U., Friederici, A., & von Cramon, D. (2002). Non-invasive regime for language lateralization in right- and left-handers by means of functional MRI and dichotic listening. *Experimental Brain Research, 145*(2), 166-176. doi:10.1007/s00221-002-1090-0
25. Hund-Georgiadis, M., Lex, U., & von Cramon, D. (2001). Language dominance assessment by means of fMRI: Contributions from task design, performance, and stimulus modality. *Journal of Magnetic Resonance Imaging, 13*(5), 668-675. doi:10.1002/jmri.1094
26. Hunter, Z. R., & Brysbaert, M. (2008). Visual half-field experiments are a good measure of cerebral language dominance if used properly: Evidence from fMR1. *Neuropsychologia, 46*(1), 316-325. doi:10.1016/j.neuropsychologia.2007.07.007
27. Jansen, A., Menke, R., Sommer, J., Foerster, A. F., Bruchmann, S., Hempleman, J., et al. (2006). The assessment of hemispheric lateralization in functional MRI - robustness and reproducibility. *Neuroimage, 33*(1), 204-217. doi:10.1016/j.neuroimage.2006.06.019
28. Jensen-Kondering, U. R., Ghobadi, Z., Wolff, S., Jansen, O., & Ulmer, S. (2012). Acoustically presented semantic decision-making tasks provide a robust depiction of the temporo-parietal speech areas. *Journal of Clinical Neuroscience, 19*(3), 428-433. doi:10.1016/j.jocn.2011.04.038
29. Kennan, R., Kim, D., Maki, A., Koizumi, H., & Constable, R. (2002). Non-invasive assessment of language lateralization by transcranial near infrared optical topography and functional MRI. *Human Brain Mapping, 16*(3), 183-189. doi:10.1002/hbm.10039
30. Kleinhans, N. M., Mueller, R., Cohen, D. N., & Courchesne, E. (2008). Atypical functional lateralization of language in autism spectrum disorders. *Brain Research, 1221*, 115-125. doi:10.1016/j.brainres.2008.04.080
31. Knecht, S., Jansen, A., Frank, A., van Randenborgh, J., Sommer, J., Kanowski, A., et al. (2003). How atypical is atypical language dominance? *Neuroimage, 18*(4), 917-927. doi:10.1016/S1053-8119(03)00039-9
32. Krainik, A., Lehéricy, S., Duffau, H., Capelle, L., Chainay, H., Cornu, P., et al. (2003). Postoperative speech disorder after medial frontal surgery: Role of the supplementary motor area. *Neurology, 60*(4), 587-594. doi:10.1212/01.WNL.0000048206.07837.59
33. Lohmann, H., Deppe, M., Jansen, A., Schwindt, T., & Knecht, S. (2004). Task repetition can affect functional magnetic resonance imaging-based measures of language lateralization and lead to pseudoincreases in bilaterality. *Journal of Cerebral Blood Flow and Metabolism, 24*(2), 179-187. doi:10.1097/01.WCb.0000100066.36077.91
34. Mazoyer, B., Zago, L., Jobard, G., Crivello, F., Joliot, M., Perchey, G., et al. (2014). Gaussian mixture modeling of hemispheric lateralization for language in a large sample of healthy individuals balanced for handedness. *Plos One, 9*(6), e101165. doi:10.1371/journal.pone.0101165
35. Miro, J., Ripolles, P., Lopez-Barroso, D., Vila-Ballo, A., Juncadella, M., de Diego-Balaguer, R., et al. (2014). Atypical language organization in temporal lobe epilepsy revealed by a passive semantic paradigm. *Bmc Neurology, 14*, 98. doi:10.1186/1471-2377-14-98
36. Morrison, M. A., Churchill, N. W., Cusimano, M. D., Schweizer, T. A., Das, S., & Graham, S. J. (2016). Reliability of task-based fMRI for preoperative planning: A test-retest study in brain tumor patients and healthy controls. *Plos One, 11*(2), e0149547. doi:10.1371/journal.pone.0149547
37. Nadkarni, T. N., Andreoli, M. J., Nair, V. A., Yin, P., Young, B. M., Kundu, B., et al. (2015). Usage of fMRI for pre-surgical planning in brain tumor and vascular lesion patients: Task and statistical threshold effects on language lateralization. *Neuroimage-Clinical, 7*, 415-423. doi:10.1016/j.nicl.2014.12.014
38. Niskanen, E., Kononen, M., Villberg, V., Nissi, M., Ranta-aho, P., Saisanen, L., et al. (2012). The effect of fMRI task combinations on determining the hemispheric dominance of language functions. *Neuroradiology, 54*(4), 393-405. doi:10.1007/s00234-011-0959-7
39. Ocklenburg, S., Hugdahl, K., & Westerhausen, R. (2013). Structural white matter asymmetries in relation to functional asymmetries during speech perception and production. *Neuroimage, 83*, 1088-1097. doi:10.1016/j.neuroimage.2013.07.076
40. Orellana, C. M., Visch-Brink, E., Vernooij, M., Kalloe, S., Satoer, D., Vincent, A., et al. (2015). Crossed cerebrocerebellar language lateralization: An additional diagnostic feature for assessing atypical language representation in presurgical functional MR imaging. *American Journal of Neuroradiology, 36*(3), 518-524. doi:10.3174/ajnr.A4147
41. Partovi, S., Jacobi, B., Rapps, N., Zipp, L., Karimi, S., Rengier, F., et al. (2012). Clinical standardized fMRI reveals altered language lateralization in patients with brain tumor. *American Journal of Neuroradiology, 33*(11), 2151-2157. doi:10.3174/ajnr.A3137
42. Partovi, S., Konrad, F., Karimi, S., Rengier, F., Lyo, J. K., Zipp, L., et al. (2012). Effects of covert and overt paradigms in clinical language fMRI. *Academic Radiology, 19*(5), 518-525. doi:10.1016/j.acra.2011.12.017
43. Perlaki, G., Horvath, R., Orsi, G., Aradi, M., Auer, T., Varga, E., et al. (2013). White-matter microstructure and language lateralization in left-handers: A whole-brain MRI analysis. *Brain and Cognition, 82*(3), 319-328. doi:10.1016/j.bandc.2013.05.005
44. Pravata, E., Sestieri, C., Mantini, D., Briganti, C., Colicchio, G., Marra, C., et al. (2011). Functional connectivity MR imaging of the language network in patients with drug-resistant epilepsy. *American Journal of Neuroradiology, 32*(3), 532-540. doi:10.3174/ajnr.A2311
45. Propper, R. E., O'Donnell, L. J., Whalen, S., Tie, Y., Norton, I. H., Suarez, R. O., et al. (2010). A combined fMRI and DTI examination of functional language lateralization and arcuate fasciculus structure: Effects of degree versus direction of hand preference. *Brain and Cognition, 73*(2), 85-92. doi:10.1016/j.bandc.2010.03.004
46. Ramsey, N., Sommer, I., Rutten, G., & Kahn, R. (2001). Combined analysis of language tasks in fMRI improves assessment of hemispheric dominance for language functions in individual subjects. *Neuroimage, 13*(4), 719-733. doi:10.1006/nimg.2000.0722
47. Razafimandimby, A., Maiza, O., Herve, P., Lecardeur, L., Delamillieure, P., Brazo, P., et al. (2007). Stability of functional language lateralization over time in schizophrenia patients. *Schizophrenia Research, 94*(1-3), 197-206. doi:10.1016/j.schres.2007.04.011
48. Ruff, I. M., Brennan, N. M. P., Peck, K. K., Hou, B. L., Tabar, V., Brennan, C. W., et al. (2008). Assessment of the language laterality index in patients with brain tumor using functional MR imaging: Effects of thresholding, task selection, and prior surgery. *American Journal of Neuroradiology, 29*(3), 528-535. doi:10.3174/ajnr.A0841
49. Rutten, G., Ramsey, N., van Rijen, P., & van Veelen, C. (2002). Reproducibility of fMRI-determined language lateralization in individual subjects. *Brain and Language, 80*(3), 421-437. doi:10.1006/brln.2001.2600
50. Sanjuan, A., Bustamante, J., Forn, C., Ventura-Campos, N., Barros-Loscertales, A., Martinez, J., et al. (2010). Comparison of two fMRI tasks for the evaluation of the expressive language function. *Neuroradiology, 52*(5), 407-415. doi:10.1007/s00234-010-0667-8
51. Sanjuan, A., Forn, C., Ventura-Campos, N., Rodriguez-Pujadas, A., Garcia-Porcar, M., Belloch, V., et al. (2010). The sentence verification task: A reliable fMRI protocol for mapping receptive language in individual subjects. *European Radiology, 20*(10), 2432-2438. doi:10.1007/s00330-010-1814-7
52. Seghier, M., Lazeyras, F., Pegna, A., Annoni, J., Zimine, I., Mayer, E., et al. (2004). Variability of fMRI activation during a phonological and semantic language task in healthy subjects. *Human Brain Mapping, 23*(3), 140-155. doi:10.1002/hbm.20053
53. Seghier, M. L., Kherif, F., Josse, G., & Price, C. J. (2011). Regional and hemispheric determinants of language laterality: Implications for preoperative fMRI. *Human Brain Mapping, 32*(10), 1602-1614. doi:10.1002/hbm.21130
54. Sepeta, L. N., Berl, M. M., Wilke, M., You, X., Mehta, M., Xu, B., et al. (2016). Age-dependent mesial temporal lobe lateralization in language fMRI. *Epilepsia, 57*(1), 122-130. doi:10.1111/epi.13258
55. Somers, M., Neggers, S. F., Diederen, K. M., Boks, M. P., Kahn, R. S., & Sommer, I. E. (2011). The measurement of language lateralization with functional transcranial doppler and functional MRI: A critical evaluation. *Frontiers in Human Neuroscience, 5*, 31. doi:10.3389/fnhum.2011.00031
56. Sommer, I., Ramsey, N., Mandl, R., & Kahn, R. (2003). Language lateralization in female patients with schizophrenia: An fMRI study. *Schizophrenia Research, 60*(2-3), 183-190. doi:10.1016/S0920-9964(02)00300-6
57. Stippich, C., Mohammed, J., Kress, B., Hahnel, S., Gunther, J., Konrad, F., et al. (2003). Robust localization and lateralization of human language function: An optimized clinical functional magnetic resonance imaging protocol. *Neuroscience Letters, 346*(1-2), 109-113. doi:10.1016/S0304-3940(03)00561-5
58. Suarez, R. O., Whalen, S., O'Shea, J. P., & Golby, A. J. (2008). A surgical planning method for functional MRI assessment of language dominance: Influences from threshold, region-of-interest, and stimulus mode. *Brain Imaging and Behavior, 2*(2), 59-73. doi:10.1007/s11682-007-9018-8
59. Sveller, C., Briellmann, R. S., Saling, M. M., Lillywhite, L., Abbott, D. F., Masterton, R. A. J., et al. (2006). Relationship between language lateralization and handedness in left-hemispheric partial epilepsy. *Neurology, 67*(10), 1813-1817. doi:10.1212/01.wnl.0000244465.74707.42
60. Szaflarski, J. P., Holland, S. K., Jacola, L. M., Lindsell, C., Privitera, M. D., & Szaflarski, M. (2008). Comprehensive presurgical functional MRI language evaluation in adult patients with epilepsy. *Epilepsy & Behavior, 12*(1), 74-83. doi:10.1016/j.yebeh.2007.07.015
61. Tailby, C., Weintrob, D. L., Saling, M. M., Fitzgerald, C., & Jackson, G. D. (2014). Reading difficulty is associated with failure to lateralize temporooccipital function. *Epilepsia, 55*(5), 746-753. doi:10.1111/epi.12607
62. Thivard, L., Hombrouck, J., du Montcel, S., Delmaire, C., Cohen, L., Samson, S., et al. (2005). Productive and perceptive language reorganization in temporal lobe epilepsy. *Neuroimage, 24*(3), 841-851. doi:10.1016/j.neuroimage.2004.10.001
63. Tie, Y., Suarez, R. O., Whalen, S., Radmanesh, A., Norton, I. H., & Golby, A. J. (2009). Comparison of blocked and event-related fMRI designs for pre-surgical language mapping. *Neuroimage, 47*, T107-T115. doi:10.1016/j.neuroimage.2008.11.020
64. Tzourio-Mazoyer, N., Marie, D., Zago, L., Jobard, G., Perchey, G., Leroux, G., et al. (2015). Heschl's gyrification pattern is related to speech-listening hemispheric lateralization: FMRI investigation in 281 healthy volunteers. *Brain Structure & Function, 220*(3), 1585-1599. doi:10.1007/s00429-014-0746-4
65. Tzourio-Mazoyer, N., Joliot, M., Marie, D., & Mazoyer, B. (2016). Variation in homotopic areas' activity and inter-hemispheric intrinsic connectivity with type of language lateralization: An FMRI study of covert sentence generation in 297 healthy volunteers. *Brain Structure & Function, 221*(5), 2735-2753. doi:10.1007/s00429-015-1068-x
66. Van der Haegen, L., Cai, Q., & Brysbaert, M. (2012). Colateralization of broca's area and the visual word form area in left-handers: FMRI evidence. *Brain and Language, 122*(3), 171-178. doi:10.1016/j.bandl.2011.11.004
67. Van der Haegen, L., Cai, Q., Seurinck, R., & Brysbaert, M. (2011). Further fMRI validation of the visual half field technique as an indicator of language laterality: A large-group analysis. *Neuropsychologia, 49*(10), 2879-2888. doi:10.1016/j.neuropsychologia.2011.06.014
68. van Oers, C. A. M. M., Vink, M., van Zandvoort, M. J. E., van der Worp, H. B., de Haan, E. H. F., Kappelle, L. J., et al. (2010). Contribution of the left and right inferior frontal gyrus in recovery from aphasia. A functional MRI study in stroke patients with preserved hemodynamic responsiveness. *Neuroimage, 49*(1), 885-893. doi:10.1016/j.neuroimage.2009.08.057
69. van Rijn, S., Aleman, A., Swaab, H., Vink, M., Sommer, I., & Kahn, R. S. (2008). Effects of an extra X chromosome on language lateralization: An fMRI study with klinefelter men (47,XXY). *Schizophrenia Research, 101*(1-3), 17-25. doi:10.1016/j.schres.2008.02.001
70. van Veelen, N. M. J., Vink, M., Ramsey, N. F., Sommer, I. E. C., van Buuren, M., Hoogendam, J. M., et al. (2011). Reduced language lateralization in first-episode medication-naive schizophrenia. *Schizophrenia Research, 127*(1-3), 195-201. doi:10.1016/j.schres.2010.12.013
71. Vassal, F., Schneider, F., Boutet, C., Jean, B., Sontheimer, A., & Lemaire, J. (2016). Combined DTI tractography and functional MRI study of the language connectome in healthy volunteers: Extensive mapping of white matter fascicles and cortical activations. *Plos One, 11*(3), e0152614. doi:10.1371/journal.pone.0152614
72. Vernooij, M. W., Smits, M., Wielopolski, P. A., Houston, G. C., Krestin, G. P., & van der Lugt, A. (2007). Fiber density asymmetry of the arcuate fasciculus in relation to functional hemispheric language lateralization in both right- and left-handed healthy subjects: A combined fMRI and DTI study. *Neuroimage, 35*(3), 1064-1076. doi:10.1016/j.neuroimage.2006.12.041
73. Vikingstad, E., George, K., Johnson, A., & Cao, Y. (2000). Cortical language lateralization in right handed normal subjects using functional magnetic resonance imaging. *Journal of the Neurological Sciences, 175*(1), 17-27. doi:10.1016/S0022-510X(00)00269-0
74. Vingerhoets, G., Alderweireldt, A., Vandemaele, P., Cai, Q., Van der Haegen, L., Brysbaert, M., et al. (2013). Praxis and language are linked: Evidence from co-lateralization in individuals, with atypical language dominance. *Cortex, 49*(1), 172-183. doi:10.1016/j.cortex.2011.11.003
75. Wilke, M., & Lidzba, K. (2007). LI-tool: A new toolbox to assess lateralization in functional MR-data. *Journal of Neuroscience Methods, 163*(1), 128-136. doi:10.1016/j.jneumeth.2007.01.026
76. Zaca, D., Jarso, S., & Pillai, J. J. (2013). Role of semantic paradigms for optimization of language mapping in clinical fMRI studies. *American Journal of Neuroradiology, 34*(10), 1966-1971. doi:10.3174/ajnr.A3628
