# Supplementary material for: Methodological considerations in assessment of language lateralisation with fMRI: a systematic review
Source: PeerJ. 2017 Jul 11;5:e3557. doi: 10.7717/peerj.3557 (PMC5508809; doi:10.7717/peerj.3557)
Supplement: Appendix S3 [file peerj-05-3557-s003.pdf]

Lateralisation

Data Exports, Reports, and Stats

VIDEO: How to use Data Exports, Reports, and Stats

Summary of selected studies

Article authors (article_authors)	Publication year (year_of_ publication)	Sample size (sample_ size)	Description of LI calculation method (li_calc_method_description)	Cut offs used for dominance classification (cut_offs_ dominance)	Specific Regions looked at (specific_regions)	Key conclusions (study_conclusions)
Abbott, Waites, Lillywhite and Jackson	2010	34	Calculated the LI at multiple thresholds, and then calculated at a threshold that yielded a fixed number of active voxels. Calculated the distribution of LI as function of the number of voxels above threshold i.e. obtained a voxel count distribution.	Data-driven method (5)	Inferior frontal and middle frontal gyri.	Laterality should not be determined at a single threshold but using thresholds that yield a fixed number of active voxels i.e. an individualy determined variable threshold, as this is more robust. Argue for the utility of an approach to dominance classification in which an LI distribution across voxel counts for a subject can be compared to a normative distribution.
Adcock, Wise, Oxbury, Oxbury, Matthews	2003	12	Four methods of calculation were compared:<br>Voxel counts, anatomically defined ROI<br>Voxel counts, functionally defined ROI<br>Signal magnitude, anatomically defined ROI<br>Signal magnitude, functionally defined ROI	Data-driven method (5)	Inferior frontal gyrus,<br>Superior temporal gyri,<br>Premotor areas,<br>Anterior cingulate gyrus	fMRI LIs are highly reproducible. Magnitude LIs were more robust than extent LIs in terms of lower variability across testing sessions. ROI had little effect on strength of LI.
Baciu, Juphard, Cousin, Le Bas	2005	10	Compared two methods:<br>Standard LI method: LIs calculated based on voxel counts within a VOI consisting of inferior frontal, temporal and parietal cortex.<br>Flip method: First, two sets of images are created; right side images (normalised L and R) and a mirror images set (flipped so that LH is on the right). Calculated the following statistical contrast: [task vs control] for right side images versus [task vs control] for mirror images. Then calculate LIs as normal using voxel counts in new images within ROI.	0.2 (2)	Inferior frontal cortex (44, 45, 47)<br>Temporal cortex (22, 21, 37)<br>Parietal cortex (40, 39)	The rhyme detection task appears more robust than the word generation task in that LI values were more resistant to the effects of normalisation, smoothing and clustering. For rhyme detection, FM appeared superior to LIM in terms of correlation with manual LIs; neither method correlated with manual LIs for word generation.
Bethmann, Tempelmann, Bleser, Scheich, Brechmann	2007	30	Used the standard LI equation using both the BOLD signal intensity and the number of activated voxels. Calculated for each region and then an average taken across regions.	0.2 (2)	Inferior frontal sulcus,<br>Inferior frontal gyrus,<br>Superior temporal sulcus,<br>Angular gyrus	Classifying language dominance based on a single ROI is not always appropriate for characterising an individual's pattern of language laterality, in light of cases of crossed regional dominance. LI extent and magnitude measures did not differ significantly in magnitude and were strongly correlated.
Berl, Zimmaro, Khan, Dustin, Ritzl, Duke et al.	2014	118	Used Wilke and Schmithorst's (2006) bootrapping method within LI toolbox.	Data-driven method (5)	Broca's area,<br>Wernicke's area	A data-driven clustering method indicated the existence of meaningful categories of language dominance versus the arbitrary categorisation cut-offs traditionally used. Laterality should be assessed on a regional rather than a global level.
Fernandez, de Greiff, von Oertzen, Reuber, Lun, Klaver et al.	2001	12	Used a variable threshold for each subject set at half the mean maximum t-value, defined as the mean of those 5% voxels showing the highest level of activation. Then calculated a weighted LI using the sum of t-values of supra-threshold voxels multiplied by the set of activated voxels. Compared two methods of LI calculation; online and offline. Laterality raters viewed intermixed real-time and offline datasets and rated them as left, right or bilateral, as well as comparing the two datasets in terms of quantified LI.	Visual inspection of contrast images (6)	Broca's area,<br>Prefrontal areas outside Broca's area,<br>Temporoparietal area	Laterality analyses based on offline and online image processing were highly correlated. For this semantic decision task, temporoparietal mean LI was higher than frontal mean LI.
Jansen, Menke, Sommer, Forster, Bruchmann, Hempleman, Weber, Knecht.	2006	10	Used the standard LI equation, and varied the choice of activity measure and definition of ROI:<br>Extent of activation (at both fixed and variable thresholds)<br>Magnitude of activation (either all within ROI, or only those exceeding a threshold level)<br>Fixed thresholds- LIs were calculated for a range of statistical thresholds.<br>Variable thresholds- threshold defined at level to yield fixed number of activated voxels (different criterion number chosen for different tasks).	0.2 (2)	Broca's area<br>Rest of prefrontal cortex<br>Temporoparietal cortex<br>Whole cerebral hemispheres (anatomical ROI)	LI extent and LI magnitude measures have similar reproducibility.<br>But, recommended that the most reproducible and robust LIs could be obtained using magnitude of signal change from those voxels exceeding a pre-set threshold value within a defined ROI.<br>The word generation task may be just as reproducible as CTA, which are both more reproducible than semantic decision.
Binder, Swanson, Hammeke, Sabsevitz	2008	26	Used the standard LI equation using voxel counts within global ROIs of the left and right hemisphere (excluding cerebellum and brainstem) at a single fixed threshold.	Dominance not classifed (4)	Global hemispheres	Resting baselines should not be used for speech comprehension protocols, due to resting activation of conceptual representations. Overall, a semantic decision- tone decision protocol was optimal in terms of producing the strongest and most consistent lateralisation.
Allendorfa, Hernando, Hossain, Nenert, Holland, Szaflarski	2016	214	Used LI toolbox on fMRI data within a functional mask. Threshold adaptively defined as the mean intensity of voxels within that mask. Number of voxels surviving this threshold in R and L hemispheres then used for standard LI calculation. Removed statistical outliers using data clustering and variance weighting options.	Other cut-offs used (3)	Single large mask including frontal, temporal and parietal regions.	Laterality strength did not differ between right and left handers for verb generation; was on average 0.44 for right handers and 0.35 for left handers.
Backes, Deblaere, Vonck, Kessels, Boon, Hofman, Wilmink et al.	2005	9	Used voxel counts within right and left hemispheres for the standard LI equation. Calculated LIs across a range of thresholds for each subject. When group comparisons were made, threshold was set at Z>3.8.	0.2 (2)	Inferior and middle frontal cortex,<br>Superior frontal cortex,<br>Anterior temporal lobe,<br>Posterior and inferior temporal lobe,<br>Temporo-parietal junction.	Strong mean LIs obtained for both word generation and text reading covert paradigms.
Brennan, Whalen, Branco, O'Shea, Norton, Golby	2007	7	Standard LI formula used (not clear if using voxel extent or magnitude), from voxels surviving threshold within ROIs. LIs were calculated at a range of thresholds.	0.2 (2)	Broca's area (inferior and middle frontal gyri),<br>Wernicke's area (superior temporal, supramarginal and angular gyri)	Automated language paradigms appear to be more weakly lateralising than non-automated speech tasks.
	2006	30	Used voxel counts within the standard LI equation for voxels surviving a single fixed threshold within each ROI independently.	Dominance not classifed (4)		Strong laterality obtained for a rhyming decision task using an active perceptual decision baseline within a frontal ROI.

Article authors (article_authors)	Publication year (year_of_ publication)	Sample size (sample_ size)	Description of LI calculation method (li_calc_method_description)	Cut offs used for dominance classification (cut_offs_ dominance)	Specific Regions looked at (specific_regions)	Key conclusions (study_conclusions)
Clements, Rimrodt, Abel, Blankner, Mostofsky, Pekar et al.					Inferior frontal gyrus,<br><br>Inferior parietal lobe.	
Cousin, Peyrun, Pichat, Lamalle, Le Bas, Baciu	2007	11	Used the flip method (Baciu et al, 2005). First, two sets of images are created; right side images (normalised L and R) and a mirror images set (flipped so that LH is on the right). Then did direct statistical comparisons between hemispheres for each task without taking into account the control condition e.g. (rhyming unflipped + non-rhyming unflipped) > (rhyming flipped + nonrhyming unflipped). Then added analyses using the flip method in which the control condition was taken into account.	Visual inspection of contrast images (6)	Frontal cortex,<br><br>Temporal cortex,<br><br>Parietal cortex,<br><br>Cerebellum,<br><br>Thalamus.	The flip method can be used to assess the significance of hemispheric asymmetries across different areas, and whether such asymmetries relate to the condition of interest or to the control task. Taking the control task into account when comparing the hemispheres yielded more left lateralised areas.
Deblaere, Backes, Hofman, Vandemaele, Boon, Vonck et al.	2002	9	Used voxel counts above a single fixed threshold within the standard LI equation.	Dominance not classifed (4)	Whole hemispheres.	Rank ordering of language tasks from strongest LI to weakest: Word generation, semantic decision, reading, naming. Confirms the strongly lateralising power of word generation, and provides some suggestions of the effects of baseline on LI; in particular, argued their baselines for the other tasks may have engaged language processes to too high an extent.
Dodoo-Schittko, Rosengarth, Doenitz, Greenlee	2012	11	Used voxel counts within ROIs for the standard LI equation. Group LIs were calculated at a single fixed threshold but also calculated LIs for each subject across a range of threshold levels to show threshold dependence.	Dominance not classifed (4)	Pars opercularis and pars triangularis,<br><br>Inferior frontal gyrus,<br><br>Superior temporal gyrus,<br><br>Angular and supramarginal gyri.	Demonstrated strong effects on laterality of both single versus combined task analysis, and resting baselines versus active control conditions. An active control task may make a STA more strongly lateralised than a CTA using resting baselines. In general, CTA yields the strongest LIs. Generation tasks yield stronger LIs than decision tasks, but the latter may be more robust against threshold dependence.
Doucet, Pustina, Skidmore, Sharan, Sperling,Tracy	2015	23	Used the LI toolbox on thresholded images, citing Wilke and Schmithorst (2006). Specific details of their calculation method not given, but reported that they compared the LIs obtained with this method with those obtained from the bootstrapping method.	Dominance not classifed (4)	Inferior frontal cortex,<br><br>Middle and superior temporal cortex.	Demonstration of strong laterality (0.59) for word generation task.
Drager, Jansen, Bruchmann, Forster, Pleger, P'zwitserlood et al.	2004	14	Used voxel counts with the standard LI equation above a single fixed threshold of p = .001.	Dominance not classifed (4)	Inferior frontal gyrus.	Overall, task difficulty had no significant effect on lateralisation within a frontal ROI for a verbal fluency task.
Fesl, Bruhns, Rau, Wiesmann, Ilmberger, Kegel et al.	2010	39	Used voxel counts within 42 ROIs to calculate LI using the standard LI equation. Calculated both at a single fixed threshold and using variable thresholds defined according to that threshold level that yielded a criterion number of above threshold voxels (7000).	Other cut-offs used (3)	Frontal cortex,<br><br>Temporal cortex,<br><br>Motor cortex,<br><br>Parieto-occipital cortex,<br><br>SMA.	This novel free reversed association task produced strong and reliable lateralisation within multiple ROIs. The most reliable LIs were obtained using a global ROI and variable (as opposed to fixed) thresholds.
Gaillard, Balsamor, Xu, Grandin, Braniecki, Papero et al.	2002	22	Used voxel counts within ROIs in the standard LI equation, at 3 different threshold levels (t = 3.0, 4.0 and 5.0).	0.2 (2)	IFG,<br><br>Middle frontal gyrus,<br><br>Wernicke's area.	The read response naming task is capable of yielding strong laterality in both frontal and temporal ROIs. This task is thus useful for lateralising temporal receptive language areas involved in reading comprehension.
Gaillard, Sachs, Whitnah, Ahmad, Balsamo, Petrella et al.	2003	22	Used voxel counts within ROIs with the standard LI equation at a threshold of t = 4.0.	0.2 (2)	Frontal lobe ROI (IFG, MFG, SFG),<br><br>IFG,<br><br>MFG,<br><br>Temporal-parietal ROI (MTG, STG, IPL).	Demonstration that semantic fluency can yield strong lateralisation particularly in frontal but also in temporal ROIs.
Haberling, Badzakova-Trajkov, Corballis	2011	60	Used the bootstrapping method (Wilke and Schitthorst, 2007) within the LI toolbox- calculates multiple LIs at multiple thresholds.	Other cut-offs used (3)	Frontal lobe.	Demonstration of strong laterality for word generation within a frontal ROI.
Haberling, Steinemann, Corballis	2016	94	Used the bootstrapping method (Wilke and Schitthorst, 2006) from LI toolbox.	0.2 (2)	Word generation: Broca's area (pars opercularis and pars triangularis)<br><br>Synonym decision: Broca's area, combined superior and middle temporal gyri.	Laterality was almost identical across ROIs and tasks, indicating that lateralization is no more pronounced for production than for comprehension. However, cases of crossed laterality were found.
Harrington, Buonocore, Farias.	2006	10	Four methods of LI calculation:<br>LI volume (voxel count) at threshold of P < .0001<br>LI volume (voxel count) at threshold of P < .001<br>LI magnitude of F statistic at threshold of P < .01<br>LI magnitude of F statistic averaged across multiple thresholds.	Dominance not classifed (4)	IFG,<br><br>Temporoparietal cortex (supramarginal, inferior parietal, middle temporal and superior temporal gyri).	Overall, the reproducibility of LIs within a given ROI depends on the task used; reproducible LIs can be found within temporal as well as frontal ROIs, provided the right task is used (e.g. story listening). Magnitude LIs were more reproducible than extent LIs. Overall, verb generation was superior in terms of strength and reproducibility of LIs across ROIs (comparable to reproducibility of CTA).
Hernandez, Andersson, Edjlali, Hommet, Cottier, Destieux et al.	2013	16	Used the flip method (Baciu et al, 2005). First, two sets of images are created for the contrast rhyming vs font matching; right side images (normalised L and R) and a mirror images set (flipped so that LH is on the right). Then did an ANOVA with 2 factors (group and hemisphere) using contrast images (rhyming vs font matching) obtained with flipped and unflipped images for both groups.<br>Then computed an LI based on the number of activated voxels in ROIs.	Dominance not classifed (4)	Inferior temporal gyrus,<br><br>Precentral gyrus,<br><br>Superior occipital gyrus,<br><br>Opercular part of IFG,<br><br>Triangularis part of IFG,<br><br>Parietal lobule.	Rhyming decision task yields significant lateralisation across multiple ROIs using the flip method of interhemispheric comparison.
	2001	14			Broca's area	

Article authors (article_authors)	Publication year (year_of_publication)	Sample size (sample_size)	Description of LI calculation method (li_calc_method_description)	Cut offs used for dominance classification (cut_offs_dominance)	Specific Regions looked at (specific_regions)	Key conclusions (study_conclusions)
Hund-Georgiadis, Lex, von Cramon.			Used number of activated pixels above a fixed threshold of Z = 5.5 within an ROI for the standard LI calculation.	Dominance not classifed (4)		Highest laterality was obtained with semantic decision compared to perceptual decision. Active baseline yielded higher LIs than passive fixation baseline. Weak laterality (i.e. bilateral activation) seen for lexical encoding task, which may be attributable to engagement of syntactic processes. No significant effect of stimulus modality, task performance, nor response hand found.
Hund-Georgiadis, Lex, Friederici, von Cramon.	2002	34	Used the number of activated pixels above a threshold of Z = 5.5 within ROIs for the standard LI equation.	Other cut-offs used (3)	Broca's area,<br><br>Superior temporal gyrus,<br><br>Whole hemisphere (global LI).	Strong laterality found for a semantic decision task vs perceptual encoding baseline across both frontal, temporal and global LIs (i.e. no effect of region). Weaker laterality found for a lexical encoding task, and for both tasks when a fixation baseline was used.
Hunter, Brysbaert.	2008	10	Used number of voxels active above threshold within ROI for the standard LI equation.	Other cut-offs used (3)	Inferior frontal cortex.	Strong laterality was yielded by the word generation task within Broca's area within a sample of left handers; weak laterality (bilaterality) was only found in 2 out of 10 subjects, with the rest showing clear and strong asymmetry.
Jensen-Kondering, Ghobadi, Wolff, Jansen, Ulmer.	2012	20	Used both voxel number and T-value of the activation within pre-defined ROIs for the standard LI equation.	Dominance not classifed (4)	Frontal speech areas- inferior and middle frontal gyrus, anterior insula.<br><br>Temporoparietal speech areas- superior and medial temporal gyri, supramarginal and angular gyri.	The strongest lateralisation within a temporal ROI was found with an auditorily presented word generation task. Strong lateralisation within temporal areas also found using visual semantic fluency and lexical decision. Overall, effect of stimulus modality and ROI was task dependent. However, across tasks and ROIs, LIs based on voxel counts were always higher than LIs based on voxel signal magnitude (t-value).
Kennan, Kim, Maki, Koizumi, Constable.	2002	6	Used the number of active voxels above a threshold of t = 1.5 for the standard LI equation.	Dominance not classifed (4)	Whole hemispheres	This sentence comprehension task requiring the detection of syntactic and semantic errors yielded relatively strong lateralisation when a global LI was used.
Kleinhans, Mueller, Cohen, Courchesne.	2008	14	Used the summed volume of significant clusters within an ROI for the following LI equation: (L-R)/ 0.5 (L+R). This yielded an LI spanning from -2 to +2.	Dominance not classifed (4)	Frontal ROI included the superior, middle and inferior frontal gyri and the insula.	Found significantly stronger lateralisation for a letter fluency task compared to a semantic fluency task using a frontal ROI; attributed to greater recruitment of right prefrontal cortex during semantic fluency.
Knecht, Jansen, Frank, van Randenborgh, Sommer, Kanowski et al.	2003	14	Used the number of active voxels above an individually defined threshold for the standard LI equation. Threshold was defined at a level yielding a fixed number of active voxels (4000).	Dominance not classifed (4)	Whole hemispheres	For word generation, no differences were found in the extent of variability in LIs between a left dominant and a right dominant group (classified by fTCD). No evidence that one group showed greater bilaterality than the other. Demonstration that some individuals do show right dominance for word generation, in a way concordant with fTCD findings.
Krinik, Lehericy, Duffau, Capelle, Chainay, Cornu, et al.	2003	6	Used the number of activated voxels surviving a fixed threshold for the standard LI equation. Separate LIs calculated using whole hemispheres and SMA as a ROI.	0.2 (2)	Whole hemispheres,<br><br>SMA.	LIs based on voxel counts within the SMA are generally weaker and more variable between individuals than LIs based on whole hemispheres, using a verbal fluency task.
Lohmann, Deppe, Jansen, Schwindt, Knecht.	2004	1	Calculated two separate LIs using the standard LI equation; one using voxel counts, one using mean signal change. The former was calculated at a fixed threshold of P = 0.001 as this was the P value at which the LI was most reproducible and most robust against moderate changes in P.	0 (1)	Global LI- whole hemispheres (excl. cerebellum).<br><br>Regional LI- areas surrounding the IFG (including Broca's area).	Task repetition led to 'pseudoincreases' in bilaterality; this was markedly demonstrated in LIs based on voxel counts. LIs based on signal magnitude showed no consistent trend over repeated testing, but were generally highly variable. Note: Based on a single subject.
Mazoyer, Zago, Jobard, Crivello, Joliot, Perchey et al.	2014	297	Used the bootstrapping method (Wilke and Schitthorst, 2006) within LI toolbox. Computed LIs based on whole hemispheres (both grey and white matter), excluding the cerebellum.	Data-driven method (5)	Whole hemispheres	A gaussian modelling approach can be used to identify dominance groups without the need for arbitrary cut-offs. 'Atypical' language laterality can be split into 'ambilateral' and 'strongly atypical' groups. Strong right hemisphere dominance was only found in left handers; otherwise, no relationship between handedness and laterality.
Miro, Ripolles, Lopez-Barroso, Vila-Ballo, Juncadella, de Diego-Balaguer et al.	2014	19	Used the bootstrapping method using voxel values (signal magnitude).	Other cut-offs used (3)	Single ROI including:<br>STG<br>MTG<br>ITG<br>Anterior temporal lobe.	This passive sentence listening task was poor at producing lateralised activity within temporal areas when compared to rest.
Morrison, Churchill, Cuimano, Schweizer, Das, Graham.	2016	12	Used the standard LI equation with both voxel counts and signal magnitude. The former was calculated across multiple thresholds to account for its threshold dependence.	0.2 (2)	Single VOI including:<br>Inferior frontal gyrus,<br><br>Superior temporal gyrus,<br><br>Angular gyrus,<br><br>Supramarginal gyrus.	Reliability of fMRI LI was task dependent. Rhyming decision laterality was more reproducible than that produced by word generation, in terms of concordance of language dominance and correlation between LI values across two testing runs.
Nadkarni, Andreoli, Nair, Yin, Young, Kundu et al.	2015	25	Used voxel counts for the standard LI equation, at four different threshold levels (t < 2.0, 2.67, 3.5, 4.0).	0.2 (2)	Broca's area,<br><br>Wernicke's area,<br><br>Other associated motor and language areas.<br><br>(See Vigneau et al's, 2006 meta-analysis).	Threshold level did not affect dominance categorisation but resulted in increasing LI values with increasing threshold levels.
Niskanen, Kononen, Villberg, Nissi, Ranta-aho, Saisanen, et al.	2012	20	The number of voxels surviving a variable threshold were used for the standard LI equation. Threshold was defined adaptively for each subject as 80% of the maximum t-value within the ROI.	Other cut-offs used (3)	Broca's area,<br><br>Wernicke's area,<br><br>Combined ROI consisting of both Broca's and Wernicke's areas, as well as BA 46, Heschl's gyrus and the hippocampus.	Overall, concluded that the optimal protocol for measuring language lateralisation with fMRI used a combined task analysis including word generation, read response naming and sentence comprehension. This combination includes both visual and auditory tasks and produced strong and consistent lateralisation.
Ocklenburg, Hugdahl, Westerhausen	2013	29	Compared both voxel count and voxel value LIs using the standard LI equation (using LI toolbox). The midline (+/-5mm) was excluded from the analysis and the threshold intensity level set to three.	Method not specified (7)	Frontal lobe,<br><br>Temporal lobe.	Strength of laterality depended largely on task and region. Strongest laterality found for word generation within frontal ROI. Reduced LI values were seen for passive speech listening compared to word generation and for temporal compared to frontal ROIs. Particularly high level of rightward asymmetry seen for passive speech listening within the temporal lobe. Correlation between the two tasks was seen for frontal but not for temporal LIs.

Article authors (article_authors)	Publication year (year_of_ publication)	Sample size (sample_ size)	Description of LI calculation method (li_calc_method_description)	Cut offs used for dominance classification (cut_offs_ dominance)	Specific Regions looked at (specific_regions)	Key conclusions (study_conclusions)
Orellana, Visch-Brink, Vernooji, Kalloe, Satoer, Vincent, et al.	2015	20	The number of voxels in each ROI was determined using a threshold-independent method (Branco's t-weighting method), and then used for the standard LI equation.	Other cut-offs used (3)	Two ROIs:<br>One cerebral ROI consisting of the IFG, superior and middle temporal gyri, and angular and supramarginal gyri.<br>One cerebellar ROI consisting of the cerebellum.	Demonstrated crossed lateralisation for cerebral versus cerebellar LIs, irrespective of whether subjects were typically or atypically lateralised for language.
Partovi, Jacobi, Rapps, Zipp, Karimi, Rengier et al.	2012b	14	Used the number of active voxels for the standard Li equation. Threshold set (variably?) at the level of the peak activation of the nondominant hemisphere.	Dominance not classifed (4)	Broca's area,<br><br>Wernicke's area (including superior temporal sulcus, middle temporal gyrus).	Strong laterality found for word generation and sentence generation tasks within frontal and temporal ROIs. Stronger laterality found in frontal than temporal ROI for word generation, but almost identical for sentence generation across ROIs.
Partovi, Konrad, Karimi, Rengier, Lyo, Zipp et al.	2012a	14	Used voxel counts for the standard LI equation. A variable threshold was used, defined as the threshold of the peak activation of the nondominant hemisphere.	Dominance not classifed (4)	Broca's area (IFG),<br><br>Wernicke's area (STG, supramarginal and angular gyri).	Covert paradigms yield stronger laterality than overt paradigms. Covert sentence and word generation tasks yield strong and highly reproducible LIs. Covert sentence generation yielded identical mean LI values within frontal and temporal ROIs, whereas covert word generation yielded stronger laterality in frontal than temporal ROIs.
Perlaki, Horvath, Orsi, Aradi, Auer, Varga et al.	2013	16	Used the bootstrapping method in LI toolbox to calculate weighted LIs averaged over multiple thresholds. Not specified whether voxel count or voxel value was used.	0.2 (2)	Frontal lobe.	Demonstration of both strong left and right lateralisation in a sample of left handers using the word generation task and a frontal ROI. 5/16 subjects were right dominant, 10/16 left dominant, 1/16 bilateral.
Pravata, Sestieri, Mantini, Briganti, Colicchio, Marra et al.	2011	12	Used voxel counts for the standard LI equation, computed across multiple thresholds between P < .164 and the least stringent P value. Computed two LIs, one global, one regional.	0.2 (2)	Two ROIs:<br><br>Global LI,<br><br>Regional LI- made up of anterior and posterior language areas.	Stronger lateralisation found for verb generation task when using a regional rather than a global LI.
Propper, O'Donnell, Whalen, Tie, Norton, Suarez et al.	2010	25	Used a threshold-independent histogram/t-weighting method (Branco et al, 2006). Involved creating histograms of number of active voxels across t thresholds, multiplying by a linear weighting function, and then integrating the area under the curve for the right and left hemispheres separately. These values were then compared in a standard LI equation format.	0 (1)	Broca's area- BA 44 and 45.<br>Wernicke's area- STG, MTG and supramarginal and angular gyri.	Demonstrated moderate laterality for an antonym generation task using a histogram method of LI calculation. Greater laterality seen for frontal (0.47) than for temporal (0.26) ROI. LI depended on handedness (but inconsistent pattern across ROIs).
Ramsey, Sommer, Rutten, Kahn.	2001	16	Used voxel counts for the standard LI equation, at two threshold levels (t = 3.0 and 4.5).	Dominance not classifed (4)	Frontal ROI- BA 44 and 45.<br>Temporal ROI- MTG, STG, angular and supramarginal gyri.<br>Frontal and temporal ROI- above areas combined.	LI was task and threshold dependent, but not ROI dependent. Overall, CTA provided the best lateralisation in terms of strength, variability/consistency and robustness against changes in threshold level. Generation tasks yielded stronger laterality than decision tasks. VG LIs were reproducible at a high but not a low threshold.
Razafimandimby, Maiza, Herve, Lecardeur, Delamillieure, Brazo et al.	2007	10	Calculated using right minus left fractional signal variation values. LI range unknown.	0 (1)	One combined ROI consisting of:<br>IFG pars triangularis,<br>MTG,<br>Angular gyrus.	Reported reproducible leftward lateralisation for passive story listening within a combined frontal and temporal ROI.
Ruff, Brennan, Peck, Hou, Tabar, Brennan, et al.	2008	7	Used voxel counts for the standard LI equation. Computed LIs at 15 different statistical thresholds. Calculated the median LI value obtained at all thresholds between P < .008 and the least stringent P value.	0.2 (2)	IFG	A verb generation task was found to be optimal for lateralisation in terms of strength and consistency of lateralization across threshold levels. Greater variability across thresholds and weaker laterality was found for semantic fluency (in some cases, dominance classification was threshold dependent).
Rutten, Ramsey, van Rijen, van Veelen	2002	9	Used voxel counts within the standard LI equation at 6 different threshold levels (from T = 2.0 to 4.5, increment 0.5).	Dominance not classifed (4)	Language ROI: IFG, IFS, posterior part of STG and MTG, supramarginal gyrus, and angular gyrus.<br><br>Global ROI: Whole hemispheres	Reproducibility of LIs depended on task and region, but was relatively independent of threshold. CTA produced the most robust and reliable lateralisation. Significantly stronger laterality was found for regional versus global LIs. Verb generation was reliable, whereas picture naming and antonym generation were not. However, strength of laterality did not differ significantly between tasks.
Sanjuan, Bustamante, Forn, Ventura-Campos, Barros-Loscertales, Martinez et al	2010	18	Used voxel counts for the standard LI equation, at a single fixed threshold.	0.2 (2)	Frontal ROI consisting of the inferior frontal gyrus and inferior frontal sulcus.	Stronger lateralisation was found for a verb generation task over a word generation task within a frontal ROI; however LIs for the tasks were significantly correlated.
Sanjuan, Forn, Ventura-Campos, Rodriguez-Pujadas, Garcia-Porcar, Belloch et al.	2010	22	Used voxel counts for the standard LI equation. Used a two-threshold (TT) correlation analysis to determine those voxels that were significantly activated within ROIs (see Auer and Frahm, 2009).	0.2 (2)	Frontal ROI: inferior frontal gyrus and sulcus.<br><br>Temporal ROI: posterior STG and MTG.<br><br>Parietal ROI: angular and supramarginal gyri.<br><br>Temporoparietal ROI: sum of the temporal and parietal ROIs.	A sentence verification task using an active phoneme decision baseline yielded strong laterality in temporoparietal areas (weaker in frontal areas). All subjects were left dominant within temporoparietal ROI (right handed sample). This is thus a suitable task for assessing lateralisation of receptive language function.
Seghier, Lazeyras, Pegna, Annoni, Zimine, Mayer, et al.	2004	26	Used voxel counts for the standard LI equation. Main LIs used a single fixed threshold of P < 0.005, but also calculated LIs across a range of threshold levels to plot TDLCs. Calculated using voxels across whole hemispheres. Also calculated a frontal dominance index, to compare laterality in frontal vs posterior ROIs: FDI = (left frontal voxels - left posterior voxels) / (left frontal voxels + left posterior voxels).	Data-driven method (5)	Global LI- whole hemispheres.<br><br>FDI- frontal and posterior ROIs in the left hemisphere.	Reported stronger and less variable laterality indices for a semantic categorisation versus a rhyming decision task. Frontal dominance indices suggested that this might be explained by a stronger frontal activation in the SC versus the RD task i.e. frontal asymmetries may be stronger for the SC versus the RD task.
Seghier, Kherif, Josse, Price	2011	82	Calculated LIs using a threshold independent approach (Nagata et al, 2001). Plots the number of L and R voxels activated across a range of threshold levels. Then calculates a non-linear regression of the curve to provide a constant term that can be used within the standard LI equation.<br><br>Also calculated voxel based laterality maps using the flip method to assess regional lateralisation. These maps code the interaction between task and hemisphere at every voxel.	0 (1)	Global LI- whole hemispheres.<br><br>Regional LI- 50 clusters across the whole brain.	The use of a global laterality index is inappropriate given regional heterogeneity in lateralisation, particularly in light of dissociations in laterality across regions, found here between the angular gyrus and ventral precentral gyrus. Indivudal variability in laterality was driven by differences in right rather than left hemisphere activation.
Sepeta, Berl, Wilke, You, Mehta, Xu, et al.	2016	57	Used the LI toolbox bootstrapping method (Wilke and Schitthorst, 2006).	0.2 (2)	MTL ROI- included hippocampi and parahippocampal gyri.<br><br>Broca's area.<br><br>Wernicke's area.	Demonstrated moderate laterality within the MTL for a sentence comprehension task, with most subjects showing left lateralisation. MTL laterality was predicted by laterality within Broca's and Wernicke's areas for the same task.

**Article authors** (article_authors)	**Publication year** (year_of_ publication)	**Sample size** (sample_ size)	**Description of LI calculation method** (li_calc_method_description)	**Cut offs used for dominance classification** (cut_offs_ dominance)	**Specific Regions looked at** (specific_regions)	**Key conclusions** (study_conclusions)
Somers, Neggers, Diederen, Boks, Kahn, Sommer.	2011	22	Used signal intensity change (measures by summing beta values) in suprathreshold voxels for the standard LI equation. Thresholds were set at the individual t values at p = 0.001.	Dominance not classifed (4)	Single mask covering the following areas:<br>IFG (pars triangularis),<br>the insula,<br>MTG,<br>STG,<br>supramarginal gyrus,<br>angular gyrus.	Demonstrated moderate laterality for the word generation task in a mixed handedness sample using a single language ROI composed of both frontal and temporal areas.
Sommer, Ramsey, Mandl, Kahn.	2003	12	Used voxel counts for the standard LI equation. Single fixed threshold used of p = 0.05 (amounts to a t value of approx. 4.5, depending on the number of voxels for each individual).	Dominance not classifed (4)	One large VOI comprising:<br>Broca's area (BA 44 and 45),<br>MTG,<br>STG,<br>Supramarginal gyrus,<br>Angular gyrus.	Demonstration of strong left lateralisation obtained for an expressive-receptive combined task analysis in a sample of female right handers.
Stippich, Mohammed, Kress, Hahnel, Gunther, Konrad, et al.	2003	14	Used cluster size (voxel counts) for the standard LI equation. Threshold adaptively set at the level of the peak activation in the non-dominant hemisphere.	Dominance not classifed (4)	Broca's area,<br><br>Wernicke's area.	Demonstration of strong laterality obtained for both a semantic fluency and a sentence generation task across both frontal and temporoparietal ROIs in a sample of right handers.
Suarez, Whalen, O'Shea, Golby	2007	13	Compared two methods of LI calculation:<br>Standard LI equation- using voxel counts across a range of threshold values.<br><br>T-weighted LI- Used Branco et al's (2006) method. First plot a histogram of number of active voxels against T-score. Multiple this distribution by a weighting function that assigns higher weight to higher T-scores. Then use integrated areas for right and left hemispheres for a standard LI equation.	Other cut-offs used (3)	Whole hemispheres (global LI),<br>IFG,<br>Supramarginal gyrus,<br>Temporoparietal gyrus,<br>Precetral gyrus,<br>Middle occipital gyrus,<br>Transverse temporal gyrus.	Laterality depended on method of LI calculation and region. The effect of stimulus modality was region dependent (but generally had no effect). Hemispheric and non-language ROI LIs were highly variable and not significantly lateralised. Significant lateralisation found in IFG and supramarginal gyrus but not in temporoparietal gyrus. SMG LI was less variable and significantly higher than IFG LI for auditory but not visual stimulus presentation.
Sveller, Briellmann, Saling, Lillywhite, Abbott, Masterton et al.	2006	70	Used voxel counts for standard LI equation, using a single fixed threshold of p < 0.001.	0.2 (2)	Broca's area (IFG),<br>Wernicke's area (posterior STG),<br>Angular gyrus,<br>Middle frontal gyrus.	Moderately strong laterality found for verb generation task in a large sample with mixed handedness using a combined language ROI. No significant relationship between handedness quotient and LI was found; however a combination of left handedness with typical lateralisation was rare (3/70 subjects).
Szaflarski, Holland, Jacola, Lindsell, Privitera, Szaflarsk.	2008	49	Used voxel counts for the standard LI equation. Used a single fixed threshold of Z score >= 2.58.	Other cut-offs used (3)	Broca's area,<br><br>Wernicke's area,<br><br>Global ROI- combined frontal and temporal ROIs.	LIs for a semantic decision task were found to be more left lateralised than those of a verb generation task. May be attributable to the use of an active tone decision baseline for SD (finger tapping used for VG). However, LIs were highly correlated between tasks, across both frontal and temporal ROIs. Frontal LIs were higher than temporal LIs.
Tailby, Weintrob, Saling, Fitzgerald, Jackson	2014	42	Method of LI calculation not specified- simply cites Abbott et al (2010).<br>Emailed author: used same method as Abbott et al.<br>Generated TDLCs for each subject plotting LI as a function of number of active voxels. Threshold of chosen LI was then defined adaptively for each subject using a set of criteria.	Dominance not classifed (4)	Inferior frontal gyrus,<br>Lingual gyrus,<br>Temporo-occipital cortex (MTG, MTS, lateral occipital cortex),<br>Medial cerebellum.<br>(Not clear if LIs calculated across all areas, or within temporo-occipital cortex only).	Strong and comparable laterality found for verb generation and word generation tasks; however precise ROI used not known.
Thivard, Hombrouck, du Montcel, Delmaire, Cohen, Samson	2005	17	Used voxel counts for the standard LI equation, at a single fixed threshold of P < 0.001.	0.2 (2)	Frontal ROI,<br><br>Temporal ROI.	LI depended on task and ROI. Strongest lateralisation found for story listening in the frontal lobes; but reported that the frontal lobes were inconsistently activated for story listening. The next strongest laterality was found for the fluency task in both frontal and temporal lobes, then story listening in temporal lobes. Sentence repetition was poorly lateralised across both ROIs.
Tie, Suarez, Whalen, Radmanesh, Norton, Golby	2009	6	Used voxel counts for the standard LI equation, at a single fixed threshold of p < 0.05.	0 (1)	Inferior frontal gyrus,<br><br>Posterior part of the superior temporal gyrus.	High degree of discordance between the statistical maps generated by event-related and block designs. Generally, the event-related paradigm yielded more activation within language areas. Difficult to draw conclusions about which gave strongest laterality due to high level of individual variability. Noted that the optimal threshold required by each design may be different.
Tzourio-Mazoyer, Marie, Zago, Jobard, Perchey, Leroux.	2015	281	Used the bootstrapping method (Wilke and Schitthorst, 2006) to calculate weighted hemispheric lateralization indices (HFLIs).	Dominance not classifed (4)	Whole hemispheres.	Significant right lateralisation found for group mean LI using a speech listening task, global ROI and an event-related design. Explained by high level of individual variability in LI (range: -70 to 80). Lateralisation depended on activity within phonological areas that had strong and opposite asymmetries
Van der Haegen, Cai, Brysbaert.	2012	57	Used Wilke and Schmithorst's (2006) bootstrapping method within LI toolbox, that calculated LI values iteratively (100 samples) at 20 threshold levels. A weighted overall mean LI was then calculated by assigning higher weight to higher thresholds. Note that a blocked design was used for word generation, but an event-related design was used for lexical decision.	Other cut-offs used (3)	IFG (pars opercularis and pars triangularis) for word generation.<br><br>Ventral occipital cortex for lexical decision task.	Demonstration of colateralization between IFG (during word generation) and vOT (during lexical decision) in the majority of subjects in a left handed sample. However, reported a small number of cases (3/57) with crossed dominance.
Van der Haegen, Cai, Seurinck, Brysbaert	2011	50	Used the bootstrapping method (Wilke and Schitthorst, 2006) within LI toolbox, to calculate LIs at 20 different threshold levels with 100 bootstrap resamples (resample ratio k = 0.25). Used this to calculated a weighted mean LI for each subject.	Other cut-offs used (3)	6 different frontal ROIs:<br>IFG pars triangularis and pars opercularis together,<br>Pars triangularis,<br>Pars opercularis,<br>Pars orbitalis,<br>Insula,<br>Precentral cortex	Strongest laterality found for a word generation task within IFG ROI (both pars opercularis and pars triangularis). Weakest laterality found within the insula. Equal numbers of typical (left) and atypical (right and bilateral) lateralised subjects in this left handed sample (50:50 ratio).
van Oers, Vink, van Zandvoort, van der Worp, de Haan, Kappelle.	2010	13	Used voxel counts for the standard LI equation.	Dominance not classifed (4)	IFG,<br><br>Posterior ROI (angular, supramarginal, superior and middle temporal gyri),<br><br>Combined frontal and posterior ROI.	Similar moderately strong laterality found across all tasks and ROIs. Strongest laterality found for picture-word matching in temporal ROI; weakest found for semantic decision in temporal ROI. Greatest difference between posterior and frontal LIs found for verb generation task (0.41 versus 0.47).
van Rijn, Aleman, Swaab, Vink, Sommer, Kahn.	2008	14	Used voxel counts for the standard LI equation, at a threshold of p < 0.001. Also used cluster thresholding, in which only clusters of 5 voxels or more were included in the voxel counts for the LI equation.	Dominance not classifed (4)	Broca's area,<br><br>Superior temporal gyrus,	Demonstration of strong laterality found across anterior and posterior language areas for a CTA consisting of verb and antonym generation and semantic decision. Strongest and least variable laterality found in angular gyrus.

**Article authors** (article_authors)	**Publication year** (year_of_ publication)	**Sample size** (sample_ size)	**Description of LI calculation method** (li_calc_method_description)	**Cut offs used for dominance classification** (cut_offs_ dominance)	**Specific Regions looked at** (specific_regions)	**Key conclusions** (study_conclusions)
					Middle temporal gyrus,<br><br>Angular gyrus,<br><br>Supramarginal gyrus.	
van Veelen, Vink, Ramsey, Sommer, van Buuren, Hoogendam.	2011	43	Used voxel counts for the standard LI equation. Threshold was defined on an individual basis using half the mean maximum t-value (Fernandez et al, 2001; Jansen et al, 2006). Calculated the mean activation value in those 5% of voxels showing the highest level of activation in that ROI; threshold set at 50% of that average maximum activation value.	Dominance not classifed (4)	Insula,<br>MTG,<br>*STG,<br>Supramarginal gyrus,<br>Angular gyrus,<br>*IFG (pars triangularis)<br><br>*LI values only reported for STG and IFG.	Found weak laterality across both a frontal and temporal ROI for a CTA consisting of verb generation, antonym generation and semantic decision in a sample of right handers, using standard LI equation with a variable threshold.
Vassal, Schneider, Boutet, Jean, Sontheimer, Lemaire.	2016	20	Used voxel counts for the standard LI equation, at a single fixed threshold of $p < 0.05$ (with minimum cluster size of 2).	Dominance not classifed (4)	Whole hemispheres.	Found strong lateralisation for a sentence comprehension task involving both semantic and syntactic processing using a global LI.
Vikingstad, George, Johnson, Cao.	2000	23	Used voxel counts (volume of activated tissue) for the standard LI equation at a single fixed threshold of $P < .0006$ (reflects both voxel thresholding and cluster thresholding).	Other cut-offs used (3)	Frontal ROI (inferior and middle frontal gyri),<br><br>Temporal ROI (supramarginal, angular and superior temporal gyri).	Moderately strong lateralisation found for verb generation and picture naming tasks. Strongest lateralisation found for verb generation in frontal ROI. Biggest difference between frontal and posterior LIs found for VG. Reported two cases of 'dissociated dominance' involving left lateralisation in one task but bilateral activity in the other (using temporal ROI for naming but frontal ROI for VG).
Vernooji, Smits, Wielopolski, Houston, Krestin, van der Lugt.	2007	20	Used voxel counts for the standard LI equation, at a fixed threshold of $p < 0.05$.	Other cut-offs used (3)	Combined frontal and temporoparietal ROI consisting of:<br>IFG,<br>Middle and superior temporal gyri,<br>Supramarginal and angular gyri.	Strength of laterality for verb generation task depended on ROI and handedness; stronger laterality seen for frontal ROIs and for right handers (LI = 0.87). Higher variability in LI values seen for left handers compared to right handers.
Vingerhoets, Alderweireldt, Vandemaele, Cai, Van der Haegen, Brysbaert et al.	2013	20	Used voxel counts for LI equation, in which R and L were the other way around i.e. $(R - L)/(R + L)$. Thus, +1 indicated total right dominance. Used variable threshold adaptively defined at that level to yield the 10% most active voxels within that ROI for each participant.	Other cut-offs used (3)	IFG,<br>Dorsolateral prefrontal cortex,<br>Ventral premotor cortex,<br>Dorsal premotor cortex,<br>SMA,<br>Posterior parietal cortex.	Strongest laterality found for a word generation task within a dorsal premotor cortex region, lowest found within posterior parietal cortex. Found some cases in which subjects showed discrepant/incongruous dominance in one ROI as compared to the other four. Overall, laterality across the 5 ROIs (frontal and parietal) was significantly and positively correlated.
Wilke, Lidzba.	2007	12	Compared multiple methods of LI calculation all based around the standard LI equation. Plotted lateralisation curves for each subject using both voxel count and voxel value. Also compared global and regional LIs. Lastly, compared the fixed threshold approach with an adaptive thresholding approach, in which the threshold is set at the mean intensity of the voxels in the image. Also looked at the effects of clustering and variance weighting, in which the variability in the value of a given voxel is taken into account when calculating the LI (highly variable voxels are devalued and so contribute less to the LI).	Dominance not classifed (4)	Frontal ROI,<br><br>Global ROI (excluding the midline).	Demonstrated no differences between voxel count and voxel value LIs in terms of LI strength or robustness against threshold/statistical outliers. Clustering and variance weighting greatly improved robustness of LI against these. Global LIs were unstable; regional LIs were argued to be more sensitive. Adaptive thresholding may be beneficial.
Tzourio-Mazoyer, Joliot, Marie, Mazoyer.	2016	297	Created contrast images for each subjects (language task versus control task). Then compared these contrast map results in left and right homotopic ROIs, by calculating the left minus the right difference in BOLD variation, to give an asymmetry index. Also computed HFLIs (global LIs) as in Mazoyer et al (2014)- but already reported in that previous paper. Then used an ANOVA for each ROI to identify the regional correlates of the differences in hemispheric lateralisation between the three dominance groups.	Data-driven method (5)	58 regions across frontal, temporal, parietal, occipital, subcortical and insular regions.	Found that asymmetries at a regional level were consistent with those at a global level in terms of language dominance. Typicals and strongly atypicals showed mirroring patterns of regional asymmetries; across 50 ROIs, LI was negatively correlated between groups. Differences in asymmetry between the groups were found in both language and non-language ROIs. Demonstration of strong frontal LI for sentence generation task.
Zaca, Jarso, Pillai.	2013	12	Used Branco et al's (2006) unthresholded method, in which the weighted sum of all voxel t values in the ROI are used for the standard LI equation.	Dominance not classifed (4)	IFG,<br>SFG,<br>MFG,<br>MTG,<br>STG.	Word generation was the most strongly lateralising task in the frontal ROIs. An auditory antonym pair decision task was particularly poorly lateralising in the STG and MTG.
