# Supplementary material for: Methodological considerations in assessment of language lateralisation with fMRI: a systematic review
Source: PeerJ. 2017 Jul 11;5:e3557. doi: 10.7717/peerj.3557 (PMC5508809; doi:10.7717/peerj.3557)
Supplement: Supplemental Information 1 — Flow diagram illustrating the search strategy and selection process for obtaining articles for inclusion in this review. Adapted from Moher et al. (2009). [file peerj-05-3557-s004.docx]

Records identified through database search
(n = 90)

Records screened for eligibility
(n = 90)

Records excluded
(n = 56)

Records whose citing articles were screened
(n = 34)

Records re-checked for eligibility
(n = 84)

Studies included in qualitative synthesis
(n = 76)

Records excluded
(n = 8)

**Screening**

**Included**

**Eligibility**

**Identification**

Records selected from citing articles
(n = 50)
