# Supplementary material for: Methodological considerations in assessment of language lateralisation with fMRI: a systematic review
Source: PeerJ. 2017 Jul 11;5:e3557. doi: 10.7717/peerj.3557 (PMC5508809; doi:10.7717/peerj.3557)
Supplement: Supplemental Information 2 [file peerj-05-3557-s005.doc]

| **Section/topic** | **#** | **Checklist item** | **Reported on page #** |
| --- | --- | --- | --- |
| **TITLE** | | |  |
| Title | 1 | Identify the report as a systematic review, meta-analysis, or both.  Title: Methodological considerations in assessment of language lateralisation with fMRI: a **systematic review** | 1 |
| **ABSTRACT** | | |  |
| Structured summary | 2 | Provide a structured summary including, as applicable: background; objectives; data sources; study eligibility criteria, participants, and interventions; study appraisal and synthesis methods; results; limitations; conclusions and implications of key findings; systematic review registration number.  Note that this review is registered as a protocol on open science framework, no registration number provided. | 2 |
| **INTRODUCTION** | | |  |
| Rationale | 3 | Describe the rationale for the review in the context of what is already known.  ‘*Interpretation of fMRI lateralisation research has been problematic due to a lack of standardisation of fMRI laterality protocols. Multiple arbitrary decisions must be made when calculating the L and R terms for use in the LI equation which might affect the LI value obtained (Jansen et al., 2006; Seghier, 2008). Such variability in methodology can preclude systematic study of language lateralisation.*’ | 4 |
| Objectives | 4 | Provide an explicit statement of questions being addressed with reference to participants, interventions, comparisons, outcomes, and study design (PICOS).  *‘The purpose of this review is to assess different protocols for fMRI measurement of language lateralisation used by studies published between 2000 and 2016. We aimed to (1) look at the methods used by different studies over this time period in order to consider whether the field is converging on common criteria for evaluating language lateralisation, and (2) consider evidence for the robustness and reliability of these different methods in order to make recommendations for future research in this field.’* | 5-6 |
| **METHODS** | | |  |
| Protocol and registration | 5 | Indicate if a review protocol exists, if and where it can be accessed (e.g., Web address), and, if available, provide registration information including registration number.  *‘A protocol for this review has been registered on Open Science Framework and can be found at* [*https://osf.io/hyvc4/*](https://osf.io/hyvc4/)*.’* | 6 |
| Eligibility criteria | 6 | Specify study characteristics (e.g., PICOS, length of follow-up) and report characteristics (e.g., years considered, language, publication status) used as criteria for eligibility, giving rationale.  *‘We reviewed studies of fMRI language lateralisation published between 2000 and 2016. Papers were selected if they met the following inclusion criteria: (1) the paper calculated and reported LIs for language using fMRI; (2) participants were healthy monolingual adults; and (3) if participants included both patients and healthy control groups, the data for controls were reported separately. Papers were excluded if: (1) they exclusively studied structural asymmetries, children or bilingualism; or (2) they used language tasks with non-European languages The rationale for restricting the search to studies on healthy, monolingual, adult participants was to reduce heterogeneity within our study sample.’* | 6 |
| Information sources | 7 | Describe all information sources (e.g., databases with dates of coverage, contact with study authors to identify additional studies) in the search and date last searched.  *‘We searched Web of Science for studies… This was last searched on 05/12/16.’* | 7 |
| Search | 8 | Present full electronic search strategy for at least one database, including any limits used, such that it could be repeated.  *‘We searched Web of Science for studies published between 2000 and 2016 using the following search terms: laterali* OR asymmetr* OR dominance; AND language OR reading; AND fMRI OR functional MRI OR functional magnetic resonance imaging OR functional MR OR function MRI; NOT schizophrenia; NOT development*; NOT child*; NOT bilingual*.’* | 6 |
| Study selection | 9 | State the process for selecting studies (i.e., screening, eligibility, included in systematic review, and, if applicable, included in the meta-analysis).  *‘Two independent reviewers screened the titles and abstracts of the resulting 90 papers to assess their eligibility then conducted full-text scans to determine whether the inclusion criteria were met. Selected lists were compared between reviewers and any discrepancies discussed and a mutual decision made. This yielded a total of 34 papers selected from the original 90. To ensure thorough coverage of the literature, papers citing these 34 articles were searched to look for additional articles that met criteria. From this, 50 additional papers were selected, bringing the total to 84 papers. A final search to re-check all 84 papers against search criteria identified 7 ineligible papers. During conductance of the review, a further paper was judged to not meet criteria.’* | 6-7 |
| Data collection process | 10 | Describe method of data extraction from reports (e.g., piloted forms, independently, in duplicate) and any processes for obtaining and confirming data from investigators.  *‘Information on these measures was recorded for each paper within a database on Redcap (research electronic data capture) software, using data extraction forms developed a priori.’* | 7 |
| Data items | 11 | List and define all variables for which data were sought (e.g., PICOS, funding sources) and any assumptions and simplifications made.  *‘For each paper, we recorded the following parameters relating to the protocol used: the type of fMRI design used, the activity measures used for LI calculation, the threshold level chosen, the use of global or regional LI calculation, the specific regions considered, the language and baseline tasks used, the use of a single or a combined task analysis and the task difficulty.’* | 7 |
| Risk of bias in individual studies | 12 | Describe methods used for assessing risk of bias of individual studies (including specification of whether this was done at the study or outcome level), and how this information is to be used in any data synthesis.  We did not assess risk of bias, as this review is not concerned with clinical trials and so issues of randomisation and blinding were not relevant. Instead, the goal of our review was to document which methods had been used, rather than to generalise from a homogeneous body of work. | N/A |
| Summary measures | 13 | State the principal summary measures (e.g., risk ratio, difference in means).  *‘The variable nature of the methods used and measures reported by different fMRI studies of language lateralisation does not permit performance of a meta-analysis. Instead, this review will document the range of methods used, and provide a qualitative summary of information from these studies that is relevant for our understanding of the robustness and reliability of LI measurement.’* | 8 |
| Synthesis of results | 14 | Describe the methods of handling data and combining results of studies, if done, including measures of consistency (e.g., I^2^) for each meta-analysis. | N/A |

Page 1 of 2

| **Section/topic** | **#** | **Checklist item** | **Reported on page #** |
| --- | --- | --- | --- |
| Risk of bias across studies | 15 | Specify any assessment of risk of bias that may affect the cumulative evidence (e.g., publication bias, selective reporting within studies). | N/A |
| Additional analyses | 16 | Describe methods of additional analyses (e.g., sensitivity or subgroup analyses, meta-regression), if done, indicating which were pre-specified. | N/A |
| **RESULTS** | | |  |
| Study selection | 17 | Give numbers of studies screened, assessed for eligibility, and included in the review, with reasons for exclusions at each stage, ideally with a flow diagram.  See above: Study selection. PRISMA flow diagram included (Figure 2). | 6-7 |
| Study characteristics | 18 | For each study, present characteristics for which data were extracted (e.g., study size, PICOS, follow-up period) and provide the citations.  Data extracted from each study can be found in Appendix S2, an excel spread-sheet drawn from the online database on Redcap software used to record information for each study. A summary table drawn from this database of main outcomes of interest for this paper can be found in Table S3. R scripts were used to plot different subsets of this data to show changes in use of different methods by studies over time (Figures 3, 4, 7, 9 and 12). | See Appendix S2, S3 and Figures 3, 4, 7, 9 and 12. |
| Risk of bias within studies | 19 | Present data on risk of bias of each study and, if available, any outcome level assessment (see item 12). | N/A |
| Results of individual studies | 20 | For all outcomes considered (benefits or harms), present, for each study: (a) simple summary data for each intervention group (b) effect estimates and confidence intervals, ideally with a forest plot. | N/A (see checklist item 13) |
| Synthesis of results | 21 | Present results of each meta-analysis done, including confidence intervals and measures of consistency. | N/A |
| Risk of bias across studies | 22 | Present results of any assessment of risk of bias across studies (see Item 15). | N/A |
| Additional analysis | 23 | Give results of additional analyses, if done (e.g., sensitivity or subgroup analyses, meta-regression [see Item 16]). | N/A |
| **DISCUSSION** | | |  |
| Summary of evidence | 24 | Summarize the main findings including the strength of evidence for each main outcome; consider their relevance to key groups (e.g., healthcare providers, users, and policy makers). | 28-29 |
| Limitations | 25 | Discuss limitations at study and outcome level (e.g., risk of bias), and at review-level (e.g., incomplete retrieval of identified research, reporting bias). | N/A |
| Conclusions | 26 | Provide a general interpretation of the results in the context of other evidence, and implications for future research.  *‘…here we have highlighted some key principles that emerge from the literature that should be considered in order to generate increased standardisation in fMRI laterality protocols across future studies. Increased homogeny in the methods used by different studies will enable better integration of research findings in order to draw conclusions as to the nature and correlates of language lateralisation.’*  *‘However, measurement of laterality from a single regional or global ROI can be misleading and does not capture potential regional heterogeneity. Therefore, lateralisation across frontal and temporoparietal ROIs for at least one expressive and one receptive task should be measured, to obtain a comprehensive picture of any individual’s pattern of hemispheric dominance for language. This will enable further work to investigate the significance of such regional heterogeneity in dominance; for example, are there any functional consequences of having crossed frontal-temporal language laterality? In this way, fMRI as a method of laterality measurement can provide unique insights into lateralisation at a regional level; this should be fully exploited in future research.’* | 28 and 29 |
| **FUNDING** | | |  |
| Funding | 27 | Describe sources of funding for the systematic review and other support (e.g., supply of data); role of funders for the systematic review.  *‘This work was supported by an Advanced Grant awarded by the European Research Council (project 694189 - Cerebral Asymmetry: New directions in Correlates and Etiology – CANDICE). Dorothy Bishop is funded by programme grant 082498/Z/07/Z from the Wellcome Trust.’* | 30 |

*From:*  Moher D, Liberati A, Tetzlaff J, Altman DG, The PRISMA Group (2009). Preferred Reporting Items for Systematic Reviews and Meta-Analyses: The PRISMA Statement. PLoS Med 6(7): e1000097. doi:10.1371/journal.pmed1000097

For more information, visit: **www.prisma-statement.org**.

Page 2 of 2
